# A Theoretical Model of Laser Heating Carbon Nanotubes

**DOI:** 10.3390/nano8080580

**Published:** 2018-07-28

**Authors:** Syahril Siregar, Sri Oktamuliani, Yoshifumi Saijo

**Affiliations:** Graduate School of Biomedical Engineering, Tohoku University, Sendai 980-8579, Japan; srioktamuliani@ymail.com (S.O.); saijo@tohoku.ac.jp (Y.S.)

**Keywords:** laser heating, carbon nanotubes, heat conduction equation, contrast agents, heating agents

## Abstract

We present a theoretical model of laser heating carbon nanotubes to determine the temperature profile during laser irradiation. Laser heating carbon nanotubes is an essential physics phenomenon in many aspects such as materials science, pharmacy, and medicine. In the present article, we explain the applications of carbon nanotubes for photoacoustic imaging contrast agents and photothermal therapy heating agents by evaluating the heat propagation in the carbon nanotube and its surrounding. Our model is constructed by applying the classical heat conduction equation. To simplify the problem, we assume the carbon nanotube is a solid cylinder with the length of the tube much larger than its diameter. The laser spot is also much larger than the dimension of carbon nanotubes. Consequently, we can neglect the length of tube dependence. Theoretically, we show that the temperature during laser heating is proportional to the diameter of carbon nanotube. Based on the solution of our model, we suggest using the larger diameter of carbon nanotubes to maximize the laser heating process. These results extend our understanding of the laser heating carbon nanotubes and provide the foundation for future technologically applying laser heating carbon nanotubes.

## 1. Introduction

Carbon nanotubes (CNTs) have an important role in nanomaterials due to its mechanical, thermal, electrical, optical and magnetic properties [1,2,3]. Recently significant progress has been made on the studies of CNTs application to biology and medicine [4,5]. The possible applications of CNTs in biology and medicine are drugs delivery, contrast agents, and heating agents [6,7].

According to the experiments from previous works, CNTs were potential candidate for heating agents in photothermal therapy (PTT) and also promised candidates for contrast agents in photoacoustic (PA) imaging [8,9,10,11]. The usage of agents in both PTT and PA imaging is to increase the temperature in the tissue as the center of interest during laser irradiation. Thus, in the case of PA imaging, the PA signal will be enhanced and in the case of PTT, the temperature rise during laser irradiation will be high enough to kill the cancer cells. The combination of diagnostic and therapy at the same time is called theranostics [12,13,14]. In this research, we call heating agents of PTT and contrast agents of PA imaging as theranostics agents.

The cancer cells can be destroyed by increasing its temperature to the 41–47 ${}^{\circ }$C [15]. Furthermore, the PTT using heating agents should be able to increase the temperature of cancer cells at least up to 41–47 ${}^{\circ }$C. This optimum temperature causes the cancer cells to become hyperthermic and damaged due to the destitute of blood supply [16].

CNTs were suggested for heating agents due to the high and broad optical absorption spectra in the visible as well as near-infrared (NIR) regions [17]. High optical absorption implies that CNTs absorb more incident light and converting absorbed light into heat. The broad absorption spectra of CNTs describes that the wavelength of laser to irradiate CNTs can be varying in the visible and NIR wavelength. The peak positions of the optical absorption spectra of CNT lie in the NIR region [17]. We suggest using the NIR laser to irradiate the CNTs since the penetration of NIR light into tissue are more efficient than visible light [18,19]. Correspondingly, by using the NIR laser and CNTs as heating agents, the deep tumor can be treated. Both PTT and PA imaging use visible or near-infrared (NIR) laser. Consequently, both mechanisms are laser heating process.

A theoretical simulation of laser heating CNTs in PTT has been performed in previous work. Toshiyuki Nakamiya et al. investigated the thermal analysis of CNTs film during pulsed laser heating by solving classical heat conduction equation using the finite element method [20]. However, they were not calculating the thermal analysis of the single molecule CNTs.

Even though, the experimental and theoretical results from previous works have supported the possibility of CNTs as theranostics agents, the theoretical explanation of temperature profile in the CNTs and its surrounding during laser heating process was not well described. Furthermore, the interface temperature is crucial since this temperature is in contact directly with the cancer cells or tissue. Moreover, the optimum specification of CNTs for theranostics agents based on theoretical approach was not clearly well defined.

The objective of the present work is to develop a simple theoretical model of laser heating CNTs in the microscopic point of view by using classical heat conduction equation. Based on our model, we would like to calculate the temperature profile in CNT and its surrounding during laser heating process. Appropriately, the interface temperature between CNT and cancer cells can be determined. Based on the solution of our model, we could suggest the effective specification of CNTs such as the diameter for future theranostics agents. Our model can be possibly extended for the laser heating nanotube-based materials such as double-walled CNTs, multi-walled CNTs, silicon nanotubes and boron-nitride nanotubes.

The paper is organized as follows. Section 2 describes the theoretical model of laser heating CNT and its simplification. The solution of model and discussion will be explained in Section 3. We also provide experimental results to support the CNTs as theranostic agents in Section 3. The summary and future recommendation will be given in Section 4.

## 2. Theoretical Model

**Figure 1:**
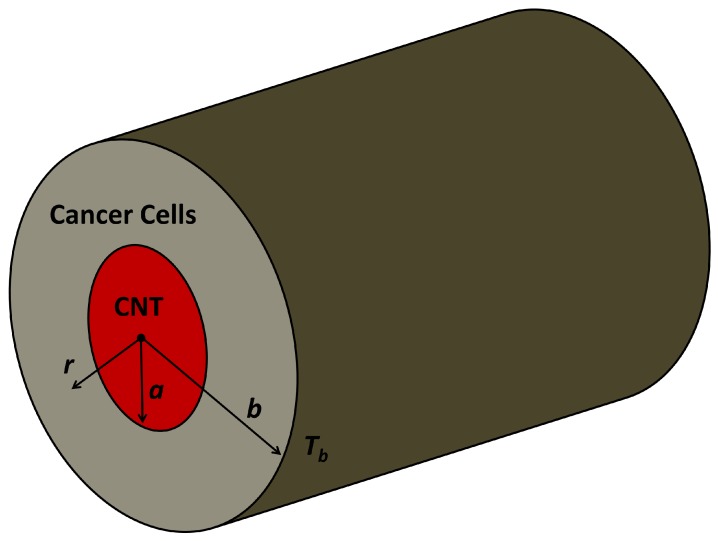
A simple theoretical model of CNT and its surrounding cancer cells during laser heating process. The CNT is modeled by a solid cylinder with the length of CNT is much larger than its diameter. The temperature on the outer side of the cylinder with distance *b* from the center $\left(T_{b}\right)$ is assumed to be the temperature of a normal human body 37 ${}^{\circ }$C.

We develop a simple model of heat propagation in the CNTs and its surrounding during laser heating process. The CNT is modeled by a solid cylinder. This assumption is common in the CNTs research, especially to study the mechanical properties of CNTs [21,22,23]. This assumption is also reasonable since the diameter of CNTs is very small and the density of atom is high especially for double-walled and multi-walled CNTs. The typical center of interest in theranostics is cancer cells. In the real case, the theranostics agents are injected into the center of interest. Consequently in our model, CNT is surrounded by cancer cells, as shown in Figure 1. The Radius of CNT is denoted by *a*. The farthest considered distance (*b*) is 100 times larger than CNT radius and its temperature ($T_{b}$) is the temperature of normal human body 37 ${}^{\circ }$C.

In order to simplify the problems, we have several assumptions. First, the length of CNT is much greater than its diameter. This assumption is reasonable since previous work reported that the length-to-diameter ratio of CNTs is around 1000 or more [24]. Therefore, the laser heating CNTs is spatially only a function of radial distance. Moreover, the laser spot is also much greater than the dimension of CNT as a consequence we can neglect the angle dependence of laser heating process.

According to our model, the laser heating CNTs can be formulated by using the classical heat conduction equation, which is the second order partial differential equation. The laser heating CNTs can be formulated as,

(1) $$\begin{array}{ccc} \rho_{c}c_{c}\frac{\partial T}{\partial t} & = & k_{c}\frac{1}{r}\frac{\partial }{\partial r}\left(r\frac{\partial T}{\partial r}\right)+q\left(r,t\right),\;\;\;\;0<r<a, \end{array}$$

(2) $$\begin{array}{ccc} \rho_{t}c_{t}\frac{\partial T}{\partial t} & = & k_{t}\frac{1}{r}\frac{\partial }{\partial r}\left(r\frac{\partial T}{\partial r}\right),\;\;\;r>a, \end{array}$$

where $\rho_{c}$ and $\rho_{t}$ are the density of CNT and cancer cells. $k_{c}$ and $k_{t}$ are the thermal conductivity of CNTs and cancer cells. *T* is temperature and *r* is radial distance measured from the center of the cylinder as shown in Figure 1. *t* is time and q(r,t) is the heating function from the laser.

We neglect the time dependence of temperature for simplicity since we would like to obtain the temperature profile as a function of radial distance. Consequently, the temperature during the laser heating process is only the function of radial distance. Further, the Equations (1) and (2) become steady-state one-dimensional conduction equation, are given by,

(3) $$\begin{array}{ccc} \frac{k_{c}}{r}\frac{d}{dr}\left(r\frac{dT}{dr}\right)+q\left(r\right) & = & 0,\;\;\;\;0<r<a, \end{array}$$

(4) $$\begin{array}{ccc} \frac{k_{t}}{r}\frac{d}{dr}\left(r\frac{dT}{dr}\right) & = & 0,\;\;\;\;r>a. \end{array}$$

Boundary and initial conditions are given by,

(5) $$\begin{array}{ccc} \frac{dT}{dr} & = & 0,\;\;\;\mathrm{at}\;\;r=0,\; \end{array}$$

(6) $$\begin{array}{ccc} {\left.k_{c}\left(\frac{dT}{dr}\right)\right|}_{r=a_{-}} & = & {\left.k_{t}\left(\frac{dT}{dr}\right)\right|}_{r=a_{+}}\; \end{array}$$

(7) $$\begin{array}{ccc} T(a_{-}) & = & T(a_{+}),\; \end{array}$$

(8) $$\begin{array}{ccc} T & = & T_{\infty }\;\left(T_{b}\right)\;\;\mathrm{at}\;r\to \infty \left(r=b\right). \end{array}$$

The temperature at the center of the CNTs during laser heating should be definable, as described in Equation (5). In Equations (6) and (7), we show that the temperature inner and outer sides of CNT must be continuous at the interface.

**Figure 2:**
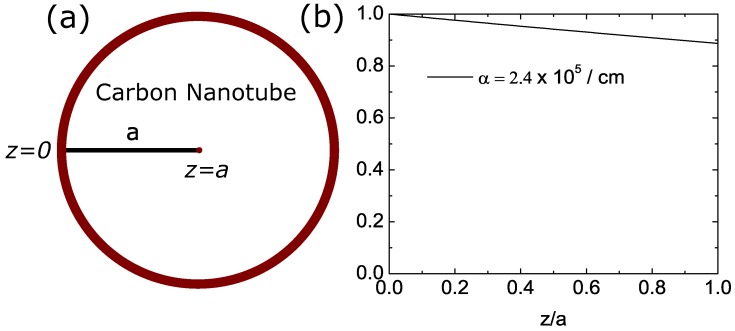
(**a**) The position of z=0 and z=a in the model measured from the interface between CNT and cancer cells. (**b**) The exponential term in the heating function with the radius of CNT is 5 nm. We assume the heating function is constant to simplify the problem.

The heating function describing heat source from the laser can be defined as,

(9) $$q\left(r\right)=\left(1-R\right)I_{0}\alpha \exp\left(-\alpha z\right),\;\;\;\mathrm{with}\;\;z=a-r.$$

where $I_{0}$ is the laser intensity, α is optical absorption coefficient of CNTs, *R* is the reflectivity since a few percent of light will be reflected by CNTs, and *z* is the depth measured from the interface to the center of the cylinder as shown in Figure 2a. The heating function is the function of radial distance *r*. The heating function will be decayed as a function of depth measured from a surface as shown in Figure 2b. However, we neglect the exponential term and assuming the heat source is constant for simplicity. This assumption seems to be reasonable since the decay of heating function is not very strong as shown in Figure 2b. This assumption is fair since in the real case, CNT is not solid cylinder, there is empty space on the inner side of the nanotube. By neglecting exponential term, the calculated temperature profile on the inner side of the cylinder is slightly higher than its expected temperature. The simplified heating function is defined as,

(10) $$q=\left(1-R\right)I_{0}\;\alpha .$$

In order to solve the heat equations, Equation (3) should be integrated. The result of integration is given by,

(11) $$\frac{dT}{dr}=-\frac{qr}{2k_{c}}+\frac{ck_{1}}{r},$$

with $ck_{1}$ is constant and its value should be zero, in order to satisfy the boundary conditions in Equation (5). Correspondingly, the Equation (11) becomes,

(12) $$\frac{dT}{dr}=-\frac{qr}{2k_{c}}.$$

The general solution for 0<r<a can be obtained by integrating Equation (12). The general solution is given by,

(13) $$T\left(r\right)=-\frac{qr^{2}}{4k_{c}}+ck_{2},$$

where $ck_{2}$ is constant. On the other hand, the solution of the heat equation in r>a region can be obtained by integrating Equation (4). The result of integration can be defined as,

(14) $$\left(\frac{dT}{dr}\right)=\frac{ct_{1}}{r},$$

with $ct_{1}$ is constant. By integrating Equation (14), we get the general solution, and is given by,

(15) $$T\left(r\right)=ct_{1}\ln r+ct_{2},$$

where $ct_{2}$ is constant. By satisfying Equation (6) in boundary condition, the $ct_{1}$ can be obtained. $ct_{1}$ can be defined as,

(16) $$\begin{array}{ccc} k_{c}\left(\frac{dT}{dr}\right) & = & k_{t}\left(\frac{dT}{dr}\right)\;\;\mathrm{at}\;r=a,\\ {\left.\frac{-qr}{2}\right|}_{r=a} & = & {\left.k_{t}\frac{ct_{1}}{r}\right|}_{r=a}\\ \frac{-qa}{2} & = & k_{t}\frac{ct_{1}}{a}\\ ct_{1} & = & -\frac{qa^{2}}{2k_{t}}. \end{array}$$

By substituting Equation (16) into Equation (15), the general solution become,

(17) $$T\left(r\right)=-\frac{qa^{2}}{2k_{t}}\ln r+ct_{2}.$$

Constant $ct_{2}$ can be formulated by substituting Equation (8) in boundary conditions into general solution in Equation (17), and defined as,

(18) $$\begin{array}{ccc} T_{b} & = & -\frac{qa^{2}}{2k_{t}}\ln b+ct_{2}\\ ct_{2} & = & T_{b}+\frac{qa^{2}}{2k_{t}}\ln b \end{array}$$

By Substituting Equation (18) into Equation (17), the real solution for r>a region can be obtained. The solution is

(19) $$\begin{array}{ccc} T(r) & = & -\frac{qa^{2}}{2k_{t}}\ln r+\frac{qa^{2}}{2k_{t}}\ln b+T_{b}\\ & = & \frac{qa^{2}}{2k_{t}}\ln\left(\frac{b}{r}\right)+T_{b} \end{array}$$

By considering the continuity from boundary conditions in Equation (7), the $ck_{2}$ can be formulated as,

(20) $$\begin{array}{ccc} T(a_{-}) & = & T(a_{+})\\ \frac{qa^{2}}{2k_{t}}\ln\left(\frac{b}{a}\right)+T_{b} & = & -\frac{qa^{2}}{4k_{c}}+ck_{2}\\ ck_{2} & = & \frac{qa^{2}}{2}\left(\frac{1}{k_{t}}\ln\left(b/a\right)+\frac{1}{2k_{c}}\right) \end{array}$$

Finally, by substituting Equation (20) into Equation (13), we can obtain the real solution for the 0<r<a region. The solution is

(21) $$\begin{array}{ccc} T(r) & = & -\frac{qr^{2}}{4k_{c}}+\frac{qa^{2}}{2k_{t}}\ln\left(\frac{b}{a}\right)+T_{b}+\frac{qa^{2}}{4k_{c}}\;\\ & = & \frac{q}{4k_{c}}\left(a^{2}-r^{2}\right)+\frac{qa^{2}}{2kt}\ln\left(\frac{b}{a}\right)+T_{b} \end{array}$$

## 3. Results and Discussion

According to the solution of our model, the temperature of CNT as a function of radial distance during laser heating process can be formulated as,

(22) $$T\left(r\right)=\left\{\begin{array}{cc} \frac{q}{4k_{c}}\left(a^{2}-r^{2}\right)+\frac{qa^{2}}{2k_{t}}\ln\left(\frac{b}{a}\right)+T_{b} & \mathrm{for}\;0\leq r\leq a,\\ \frac{qa^{2}}{2k_{t}}\ln\left(\frac{b}{r}\right)+T_{b} & \mathrm{for}\;r>a. \end{array}\right.$$

Based on Equation (22), the maximum temperature is located at the center of CNT. Then, the temperature decreases from center to the interface of CNT and cancer cells. However, the results are obtained by assuming the heating function is constant as shown in Equation (10). Consequently, the calculated temperature at the center of CNT is higher than its fact.

**Figure 3:**
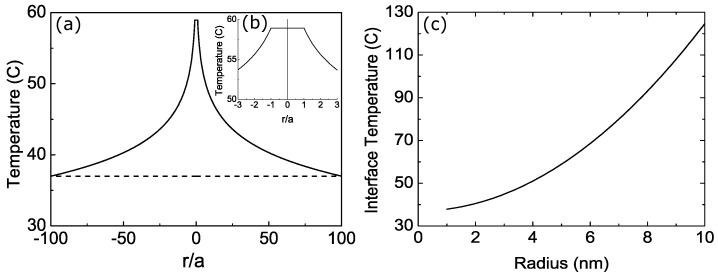
(**a**) The temperature profile of CNT and its surrounding as a function of radial distance relative to the radius of CNT during laser heating process. (**b**) The temperature profile in the regions of 0<r<a. (**c**) The interface temperature during laser heating as a function of CNT radius. The physical parameters can be seen in Table 1.

The temperature during the laser heating process is proportional to the radius of CNT (*a*) as shown in Equation (22). Thus, we suggest using the larger diameter of CNT to maximize the laser heating process in many application such as PA imaging contrast agents and PTT heating agents. We plot the temperature profile during laser heating process as shown in Figure 3a,b. We select the radius of CNT is 5 nm. The physical parameters can be seen in Table 1.

The temperature at the interface CNT and cancer cells is important because it is in contact to the cancer cells as a center of interest. The temperature at the interface as a function of CNT radius during laser heating process can be formulated as,

(23) $$T\left(a\right)=\frac{qa^{2}}{2k_{t}}\ln\left(\frac{b}{a}\right)+T_{b}.$$

The temperature at the interface is proportional to the radius of CNT as shown in Equation (23). There is also dependence to the natural logarithm of the ratio between the farthest distance (*b*) and the radius of CNT (*a*). In this research, we select the farthest distance is 100 times larger than CNT radius. In Figure 3c, we show the temperature at interface between CNT and tissue during laser heating process as a function of the radius of CNT.

## 4. Laser Heating Experiment

**Figure 4:**
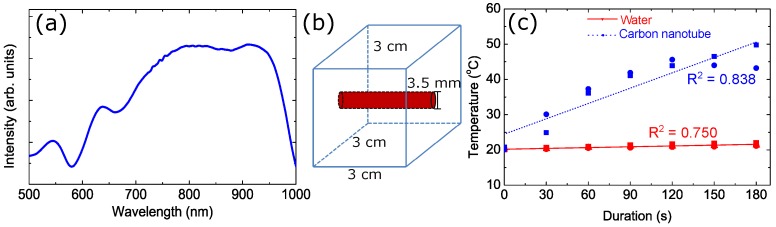
(**a**) The optical absorption of (6,5) SWNT, (**b**) the schematic diagram of the phantom for laser heating experiments, and (**c**) the temperature of the samples as a function of the laser heating duration.

We develop the experiment of laser heating to support our theoretical model. The CNT sample is commercially available CoMoCAT SWNT (6,5) powder. CNTs are not dissolve in water [29]. Consequently, we use Polyethylene glycol (PEG)-400 as a solvent. The method to prepare the sample can be found in Ref. [17]. In Figure 4a, we show the optical absorption of CNT measured using Ultraviolet-Visible (UV-VIS) spectrometer. The UV-VIS spectra confirms that CNT has strong absorption in the visible and infrared regions. The first peak is located in 500 nm–570 nm (visible) and the location of second peak is 650 nm–950 nm (visible-infrared).

The ultrasound phantom is created to describe the real case of laser heating CNT in the tissue. The ultrasound phantom is made by polyvinyl alcohol (PVA) as basic material and dimethyl sulfoxide (DMSO) as a solvent. The size of phantom is 3 cm × 3 cm × 3 cm with the tube in the center of cube as shown in Figure 4b. We put the sample in the tube. The specification of laser are pulse repetition frequency (PRF) 10 kHz, wavelength 532 nm (green), pulse duration 5.9 ns and the power of laser 2.5 Watt.

The sample is irradiated by laser and we measure the temperature of sample every 30 s in 3 min. The rise in temperature during laser irradiation can be seen in Figure 4c. The CNT has higher temperature rise than water with the gradient 0.14 ${}^{\circ }$C/s. The water has gradient 0.007 ${}^{\circ }$C/s.

## 5. Conclusions

We have successfully developed the theoretical model of laser heating CNTs by modeling the CNT as a solid cylinder. The temperature profile in CNT and its surrounding during laser heating process is obtained by solving classical steady state one-dimensional heat conduction equation. According to our calculation results, the maximum temperature during laser heating process is located at the center of CNT because we neglect the exponential term of heating function. Correspondingly, the calculated temperature inside the CNT is higher than its fact.

The temperature during laser heating process is proportional to the CNT radius. The suggested specification of CNTs for theranostic agents is the CNTs with larger diameter to maximize the laser heating process. These results bring additional understanding of the laser heating CNTs and provide the foundation for future technological application of laser heating CNTs.

Our experimental results support the ability of CNTs as theranosics agents. The gradient of CNT temperature during laser heating process is 0.14 ${}^{\circ }$C/s and the gradient temperature of water is only 0.007 ${}^{\circ }$C/s.

## Figures and Tables

**Table 1:** Physical parameters of cancer cells, CNTs and laser.

Physical Parameters		
Thermal conductivity of human tissue	$k_{t}$	0.567 W/mK [25]
Thermal conductivity of CNTs	$k_{c}$	3000−3500 W/mK [26]
Initial temperature	$T_{\infty }$	37 ${}^{\circ }$C
Reflectivity	*R*	0.1
Absorption coefficient of CNTs	α	2.4 × $10^{7}\;m^{-1}$ [27]
Laser intensity	$I_{0}$	$1\times 10^{6}\;W/{\mathrm{cm}}^{2}$ [28]
Radius of SWNT	*a*	5 nm
The farthest considered distance	*b*	100a
